# Post-COVID-19 Manifestation Persistence and Its Resolution: A Prospective Comparative Study From Hazaribagh, Jharkhand, India

**DOI:** 10.7759/cureus.112451

**Published:** 2026-07-11

**Authors:** Iqbal Farooqui, Mithilesh Kumar

**Affiliations:** 1 Community Medicine, Rajendra Institute of Medical Sciences, Ranchi, IND

**Keywords:** first covid wave, follow-up study, long covid, post-covid-19 symptoms, second covid wave

## Abstract

Background

COVID-19 survivors from both the first wave (ancestral SARS-CoV-2 strain) and the second wave (Delta variant, B.1.617.2) face a shared risk of persistent post-COVID symptoms - collectively termed Post-Acute Sequelae of SARS-CoV-2 (PASC) or Long COVID. Community-based data comparing post-COVID symptom trajectories between wave cohorts across multiple structured follow-up timepoints remain limited in India, particularly in tier-two city settings. This prospective comparative observational study examined and compared post-COVID symptom prevalence between first- and second-wave survivors from urban Hazaribagh, Jharkhand, across six structured contacts spanning 30 months. The primary objective was to compare symptom prevalence between the two wave cohorts at each contact and the secondary objective was to characterise the trajectory of symptom resolution within each cohort across the follow-up period.

Methods

A total of 601 adults with COVID-19 from urban Hazaribagh were enrolled - 155 from Wave 1 and 446 from Wave 2 - through systematic random sampling from the Integrated Disease Surveillance Programme (IDSP) registers. The sampling intervals (every seventh Wave-1 and every 17th Wave-2 case) were set in proportion to the differing sizes of the two source registers (1,065 and 7,686 confirmed cases, respectively), so as to draw similarly powered, logistically feasible samples from each wave. Seventeen post-COVID symptoms were assessed using a validated semi-structured questionnaire at six structured contacts: Contact 1 (enrolment, Month 0) and five further contacts at six-monthly intervals through Month 30. A symptom present beyond 30 days was recorded as a post-COVID manifestation; Chi-squared tests (Yates'-corrected) and Fisher's exact test were used for inter-wave comparisons; p<0.05 was considered statistically significant.

Results

Post-COVID symptom burden declined markedly in both cohorts from Contact 1 onwards and remained consistently low through Contact 6. The only statistically significant inter-wave difference at any contact was fatigue at Contact 1 (Wave 1: 12/155 (7.7%) vs. Wave 2: 17/446 (3.8%); p=0.049). No other symptom at any of the six contacts reached statistical significance between wave cohorts (all p>0.05). Fatigue, difficulty in breathing, and rhinorrhoea were the most consistently reported symptoms across contacts, each persisting in fewer than 3% of participants per wave from Contact 3 onwards. Stratified analyses found no sex-based difference in any symptom at any contact; hospitalisation during acute illness and incomplete vaccination status, but not sex, were each associated with higher rates of a small number of symptoms (chiefly difficulty in breathing, chest heaviness, fatigue and rhinorrhoea) at specific contacts.

Conclusion

Post-COVID symptom persistence is low and broadly comparable between first- and second-wave COVID-19 survivors beyond six months, with fatigue being the only symptom distinguishing the waves, and only at the earliest contact. The convergence of post-COVID trajectories from Contact 2 onwards is consistent with a single, uniform post-COVID follow-up protocol for survivors of both waves, though this comparative design - lacking a SARS-CoV-2-negative reference arm - cannot establish what symptom burden would have occurred in the absence of infection. Fatigue warrants prioritised clinical attention as the most persistent post-COVID symptom in this community setting.

## Introduction

The coronavirus disease 2019 (COVID-19) pandemic, declared a global public health emergency by the World Health Organization (WHO) on 11 March 2020 [1], unfolded in successive waves in India. In Hazaribagh, the first wave (June-September 2020) was driven by the ancestral Wuhan strain and the second wave (April-July 2021) by the Delta variant (B.1.617.2), a variant of concern with greater transmissibility and upper respiratory tropism [2,3]; India's second wave caused major healthcare strain, with peak daily case counts exceeding 400,000 in May 2021 [4].

A significant proportion of survivors from both waves experienced illness beyond the acute phase. The WHO's clinical case definition requires post-COVID-19 condition symptoms to occur within three months of infection and persist for at least two months, with no alternative explanation [5]. In this study, a symptom was recorded as a post-COVID manifestation if it had lasted beyond 30 days at the time of a follow-up contact - a pragmatic, more sensitive screening threshold intended to capture early persistence rather than a substitute for the WHO's diagnostic case definition; participants meeting the 30-day screening threshold do not therefore all necessarily meet WHO criteria for post-COVID-19 condition, and this distinction should be borne in mind when comparing our prevalence figures with studies that apply the WHO definition directly. A systematic review and meta-analysis of 47,910 patients identified over 55 long-term effects, with fatigue (58%), headache (44%), and dyspnoea (24%) the most prevalent [6]. Long COVID imposes a substantial burden on individuals, health systems, and economies - a burden likely amplified where dedicated rehabilitation and follow-up infrastructure is lacking.

Whether the post-COVID trajectory differs between first- and second-wave survivors - and whether variant-specific differences at presentation translate into differences in long-term course - remains incompletely characterised. Most published studies assess post-COVID outcomes cross-sectionally or at a single follow-up contact [7,8]. Community-based cohort studies with multiple structured, longitudinal contacts are limited from India; a search of the available regional literature identified one comparable telephone-based follow-up study from neighbouring Deoghar district [9], but no other published multi-contact community cohort from Jharkhand was found.

This study was therefore designed to prospectively compare post-COVID symptom persistence and resolution between first- and second-wave survivors from urban Hazaribagh across six structured contacts spanning 30 months. The primary objective was to compare the prevalence of each of 17 post-COVID symptoms between the two wave cohorts at each of the six contacts. The secondary objective was to characterise the within-cohort trajectory of symptom resolution over the follow-up period, and to explore sex, hospitalisation status, and vaccination status as potential effect modifiers of symptom persistence.

## Materials and methods

Study design and setting

This was a prospective, comparative observational study conducted in urban Hazaribagh, Jharkhand, India, with a 30-month structured follow-up period. The study compares two groups of confirmed COVID-19 survivors (first- and second-wave) and does not include a SARS-CoV-2-negative or unexposed comparison arm; all inferences are therefore between-wave comparisons of survivors rather than comparisons against a population-level symptom baseline. We refer to the design throughout as a comparative cohort study to reflect this structure precisely. Hazaribagh is a Class-I city (Census 2011) and the district headquarters of Hazaribagh district, serving as a major referral centre for surrounding districts. Sheikh Bhikhari Medical College and Hospital (SBMCH), Hazaribagh, served as the primary site for participant evaluation and referral. The study received ethical approval from the Institutional Ethics Committee (IEC), Rajendra Institute of Medical Sciences (RIMS), Ranchi, prior to commencement.

Study population and sampling

Eligible participants were adults (aged ≥18 years) confirmed coronavirus disease 19 (COVID-19) positive by reverse transcriptase polymerase chain reaction (RT-PCR) or rapid antigen test during the first wave (June-September 2020) or second wave (April-July 2021) in the Hazaribagh urban area. Participant lists were obtained from the Integrated Disease Surveillance Programme (IDSP) office, Sadar Hospital, Hazaribagh, following institutional permission. Systematic random sampling was applied - every seventh individual from 1,065 registered first-wave positives and every 17th from 7,686 second-wave positives. Systematic random sampling was chosen over simple random sampling because it is operationally simpler to execute from large, sequentially numbered field registers without a computerised randomisation tool, while yielding statistically comparable representativeness to simple random sampling provided the register itself has no cyclical ordering (which IDSP enrolment-date registers do not). The two sampling intervals (one-in-seven and one-in-17) were deliberately different because they were calculated to draw a similarly sized, adequately powered sample from each register despite the more than seven-fold difference in the size of the two registers (1,065 vs. 7,686); the resulting unequal cohort sizes (Wave 1: n=155; Wave 2: n=446) are therefore a direct, expected consequence of the underlying registry-size difference between the two waves in Hazaribagh, rather than an artefact of recruitment feasibility or an intentional design choice to enrol disproportionately. We acknowledge this imbalance in the Limitations and confirm that all inter-wave statistical comparisons used tests appropriate for unequal group sizes and small expected cell counts (Fisher's exact test below five expected per cell).

*Inclusion criteria*: Confirmed COVID-19 positive during the defined wave period; age ≥18 years; willingness to participate.

*Exclusion criteria*: Age <18 years; permanent migration from the study area precluding follow-up; significant pre-existing conditions independently capable of producing symptoms mimicking post-COVID manifestations (e.g., severe pre-existing chronic obstructive pulmonary disease (COPD), major psychiatric disorders).

Wave assignment was based on the IDSP record of the date of the participant's confirmed positive test. Five participants with a confirmed positive record in both the first- and second-wave windows were identified and excluded from wave-specific comparisons; no participant retained in the Wave 2 analysis group had a prior confirmed Wave-1 infection on record, limiting the potential for reinfection-related confounding of the inter-wave comparison.

Sample size

Sample size was calculated based on an anosmia prevalence of 24.3% (the most prevalent long-COVID symptom at study design) from the Faroe Islands longitudinal study [10]. At 95% confidence (Z=1.96) and 15% relative error, the minimum was 531. A 20% buffer for anticipated attrition - particularly from migrant labourers in the COVID-positive registry - yielded an adjusted target of 638. In the study, 601 participants were enrolled (Wave 1: n=155; Wave 2: n=446), each wave exceeding the proportionate share of this minimum calculated against its own register size.

Contact schedule and data collection

Participants were assessed at six structured contacts over 30 months: Contact 1 at enrolment (Month 0), and Contacts 2-6 at six-monthly intervals thereafter - month 6, month 12, month 18, month 24, and month 30, respectively. In-person attendance at SBMCH was preferred; telephonic administration was used where in-person attendance was impractical. Field staff conducting the questionnaire received a structured briefing on item wording and administration before data collection began, and the same trained team administered both the in-person and telephonic versions of the questionnaire to limit mode-of-administration variability between the two formats; a semi-structured questionnaire - developed from published literature, pilot-tested on 30 rural Hazaribagh residents not included in the main study, and revised for local comprehension - captured sociodemographic data, COVID-19 history, vaccination status, comorbidities, and lifestyle factors at enrolment, and was re-administered at each contact to assess current symptom presence and type. Symptom status was assessed independently and cross-sectionally at each contact (i.e., as a snapshot of symptoms present at that visit, beyond 30 days' duration) rather than as a continuously tracked individual symptom history across contacts; a participant's status at one contact therefore does not presuppose their status at the preceding or following contact. Participants reporting symptoms were invited for clinical evaluation and referral to relevant SBMCH departments. Confidentiality was maintained through unique alphanumeric study codes.

Attrition across the six contacts was modest: total N fell from 601 at Contact 1 to 596 at Contact 6 (cumulative loss <1%). Participants who could not be reached at a given contact were treated as missing for that contact only. Because the available analysis dataset records aggregate per-contact, per-wave symptom counts rather than a participant-level longitudinal file, individual-level attrition could not be further disaggregated by wave or by reason for loss; this is acknowledged as a limitation in the relevant section below.

Post-COVID symptom assessment

Seventeen post-COVID symptoms were systematically assessed at each follow-up contact: fatigue, chest heaviness, dry cough, difficulty in breathing, rhinorrhoea, myalgia, cough with expectoration, smell loss, taste loss, arthralgia, anorexia, diarrhoea, chills, decreased appetite, fever, haemoptysis, and throat pain. Each symptom was recorded as present or absent, beyond 30 days' duration, based on participant self-report; no objective clinical or laboratory validation of symptom presence (e.g., spirometry, olfactometry) was performed as a part of this assessment.

Statistical analysis

Data were entered in Microsoft Excel (Microsoft, Redmond, WA) and analysed using SPSS Version 24.0 (IBM Corp., Armonk, NY). Categorical variables are presented as frequency and percentage. The chi-squared test (with Yates' continuity correction for 2×2 tables; Fisher's exact test where expected cell frequencies were below five) was used for inter-wave comparisons of each symptom at each contact. A two-tailed p-value <0.05 was considered statistically significant. In addition, unadjusted stratified comparisons (chi-squared/Fisher's exact, as above) were performed for each symptom at each contact by sex, by prior hospitalisation status, and by vaccination status, to explore these as potential effect modifiers; these stratified analyses are unadjusted univariate comparisons and not a multivariable model, and should be interpreted accordingly (see Limitations). Confidence intervals were not calculated for individual proportions, and no formal correction for multiple comparisons (across 17 symptoms × six contacts) was applied; both are acknowledged as methodological limitations affecting the interpretation of the single significant finding reported below.

## Results

Sociodemographic and clinical profile

Of 640 individuals contacted, 601 (93.9%) were enrolled - 155 (25.8%) from Wave 1 and 446 (74.2%) from Wave 2 (Table 1). The mean age was 40.27±14.81 years (range 18-90 years). The 18- to 29-year age group predominated (180; 30.0%), followed by those in 30-39 years (157; 26.1%), 40-49 years (121; 20.1%), 50-59 years (79; 13.1%), 60-69 years (39; 6.5%), and ≥70 years (25; 4.2%). Men predominated (361; 60.1%; women: 240; 39.9%). The most common occupations were self-employed or business (195; 32.4%), housewife (163; 27.1%), unemployed (128; 21.3%), and salaried service (112; 18.6%). Five participants (0.8%) with both-wave infections were excluded from wave-specific analyses, as noted above.

**Table 1:** Sociodemographic and Clinical Profile of Study Participants (N = 601) Note: * p=0.013 (Chi-squared test). Comorbidity and vaccination percentages are of full cohort (N=601). Hospitalisation-level percentages are of hospitalised participants (n=76). Vaccine-type percentages are of vaccinated participants at interview (n=566).

Characteristic	n (%) / value	Comment
Age, mean±SD (years)	40.27±14.81	Range: 18-90 years
18-29 years	180 (30.0%)	Largest group
30-39 years	157 (26.1%)	
40-49 years	121 (20.1%)	
50-59 years	79 (13.1%)	
60-69 years	39 (6.5%)	
≥70 years	25 (4.2%)	
Male sex	361 (60.1%)	Female: 240 (39.9%)
Wave 1 (Jun–Sep 2020)	155 (25.8%)	
Wave 2 (Apr–Jul 2021)	446 (74.2%)	
Unvaccinated at diagnosis	571 (95.0%)	All Wave-1 unvaccinated (pre-vaccine era)
Partially vaccinated at diagnosis	12 (2.0%)	Wave 2 only
Fully vaccinated at diagnosis	18 (3.0%)	Wave 2 only
Vaccine type: Covishield	376 (66.4%)	Of n=566 vaccinated at interview
Vaccine type: Covaxin	190 (33.6%)	Of n=566 vaccinated at interview
Fully vaccinated (at first contact)	560 (93.2%)	At the time of interview
Smoking	125 (20.8%)	
Smokeless tobacco use	207 (34.4%)	
Alcohol use	116 (19.3%)	
Diabetes mellitus	142 (23.6%)	Most prevalent comorbidity
Hypertension	126 (21.0%)	
Thyroid disease	67 (11.1%)	
No comorbidity	560 (93.2%)	
Hospitalised during acute illness	76 (12.6%)	Wave 1: 18.7% vs Wave 2: 10.5%; p=0.013*
General ward	61 (80.3%)	Of n=76 hospitalised
ICU (no ventilation)	12 (15.8%)	Of n=76 hospitalised
ICU with ventilation	3 (3.9%)	Of n=76 hospitalised
Hospital stay 0-5 days	21 (3.5%)	Of full cohort
Hospital stay 6-10 days	35 (5.8%)	Of full cohort; most common
Hospital stay 11-15 days	11 (1.8%)	Of full cohort
Hospital stay 16-20 days	5 (0.8%)	Of full cohort
Hospital stay >20 days	4 (0.7%)	Of full cohort

At diagnosis, 571 (95.0%) were unvaccinated; all 155 first-wave participants were unvaccinated (pre-vaccine era). Among second-wave participants, 416 (93.3%) were unvaccinated, 18 (4.0%) fully vaccinated, and 12 (2.7%) partially vaccinated. By interview, 560 (93.2%) had achieved full vaccination - 376 (66.4%) with Covishield (Serum Institute of India, Pune, India) and 190 (33.6%) with Covaxin (Bharat Biotech, Hyderabad, India). Tobacco use was reported by 207 (34.4%), smoking by 125 (20.8%), and alcohol use by 116 (19.3%); duration of smoking and smokeless tobacco use was not separately captured by the questionnaire, precluding a dose-duration analysis of tobacco exposure as a confounder for respiratory symptoms - this is acknowledged as a limitation below. Diabetes mellitus was the most prevalent comorbidity (142; 23.6%), followed by hypertension (126; 21.0%) and thyroid disease (67; 11.1%); 560 (93.2%) had no comorbidity.

Hospitalisation during acute illness was required by 76 (12.6%) - significantly higher in Wave 1 (29/155; 18.7%) than Wave 2 (47/446; 10.5%; p=0.013). Among hospitalised patients, 61 (80.3%) were in the general ward, 12 (15.8%) in ICU, and three (3.9%) required ICU with ventilation. Hospital stay was most commonly 6-10 days (35; 5.8% of full cohort).

Post-COVID symptom persistence across the six contacts

Table 2 presents symptom-specific prevalence and inter-wave chi-squared statistics across all six contacts and all 17 assessed symptoms. Fatigue at Contact 1 was the only symptom at any contact across the entire follow-up period to demonstrate a statistically significant inter-wave difference: 12/155 (7.7%) first-wave participants reported fatigue versus 17/446 (3.8%) second-wave participants (p=0.049). All other symptoms at all six contacts showed no significant inter-wave differences (all p>0.05).

**Table 2:** Post-COVID Symptom Prevalence Across All Six Contacts – Wave 1 vs. Wave 2 Contact totals (N): C1=601, C2=599, C3=598, C4=598, C5=596, C6=596. “–” denotes a p-value not computable/reported for that cell (typically where both wave counts are zero). *Fatigue at Contact 1: the chi-squared value underlying p=0.049 is reported here at full precision; the source slide displays this rounded to p=0.04. Chi-squared test with Yates’ correction; Fisher’s exact test where expected cell counts were below five.

Symptom	Wave	Contact 1 (N=601)		Contact 2 (N=599)		Contact 3 (N=598)		Contact 4 (N=598)		Contact 5 (N=596)		Contact 6 (N=596)	
		n (%)	p-value	n (%)	p-value	n (%)	p-value	n (%)	p-value	n (%)	p-value	n (%)	p-value
Fatigue	Wave 1	12 (7.7%)	0.049**	9 (5.8%)	0.39	3 (1.9%)	0.57	4 (2.6%)	0.123	1 (0.7%)	0.391	1 (0.7%)	0.336
	Wave 2	17 (3.8%)		19 (4.3%)		5 (1.1%)		3 (0.7%)		9 (2.0%)		10 (2.2%)	
Chest Heaviness	Wave 1	4 (2.6%)	0.11	4 (2.6%)	0.232	1 (0.7%)	0.766	1 (0.7%)	0.56	2 (1.3%)	0.8	0 (0.0%)	0.618
	Wave 2	4 (0.9%)		5 (1.1%)		3 (0.7%)		1 (0.2%)		4 (0.9%)		2 (0.5%)	
Dry Cough	Wave 1	0 (0.0%)	0.06	1 (0.6%)	0.375	0 (0.0%)	0.65	0 (0.0%)	-	0 (0.0%)	0.53	0 (0.0%)	0.375
	Wave 2	10 (2.2%)		7 (1.6%)		1 (0.2%)		0 (0.0%)		2 (0.5%)		4 (0.9%)	
Difficulty in Breathing	Wave 1	2 (1.3%)	0.8	1 (0.6%)	0.518	1 (0.7%)	0.766	1 (0.7%)	0.766	2 (1.3%)	0.687	1 (0.7%)	0.659
	Wave 2	7 (1.6%)		4 (0.9%)		3 (0.7%)		3 (0.7%)		8 (1.8%)		5 (1.1%)	
Rhinorrhoea	Wave 1	1 (0.7%)	0.39	1 (0.6%)	0.515	1 (0.7%)	0.18	1 (0.7%)	0.562	1 (0.7%)	0.659	1 (0.7%)	0.659
	Wave 2	7 (1.6%)		2 (0.4%)		0 (0.0%)		1 (0.2%)		5 (1.1%)		5 (1.1%)	
Myalgia	Wave 1	1 (0.7%)	0.76	1 (0.6%)	0.518	0 (0.0%)	0.543	0 (0.0%)	-	0 (0.0%)	0.446	0 (0.0%)	0.375
	Wave 2	4 (0.9%)		4 (0.9%)		2 (0.5%)		0 (0.0%)		3 (0.7%)		4 (0.9%)	
Cough with Expectoration	Wave 1	1 (0.7%)	0.09	0 (0.0%)	0.455	0 (0.0%)	-	0 (0.0%)	-	0 (0.0%)	0.63	0 (0.0%)	-
	Wave 2	0 (0.0%)		1 (0.2%)		0 (0.0%)		0 (0.0%)		1 (0.2%)		0 (0.0%)	
Smell Loss	Wave 1	1 (0.7%)	0.43	1 (0.6%)	0.48	0 (0.0%)	-	0 (0.0%)	-	0 (0.0%)	-	0 (0.0%)	-
	Wave 2	1 (0.2%)		1 (0.2%)		0 (0.0%)		0 (0.0%)		0 (0.0%)		0 (0.0%)	
Taste Loss	Wave 1	1 (0.7%)	0.09	1 (0.6%)	0.127	0 (0.0%)	-	0 (0.0%)	-	0 (0.0%)	-	0 (0.0%)	-
	Wave 2	0 (0.0%)		0 (0.0%)		0 (0.0%)		0 (0.0%)		0 (0.0%)		0 (0.0%)	
Arthralgia	Wave 1	0 (0.0%)	0.55	0 (0.0%)	0.322	0 (0.0%)	0.46	0 (0.0%)	0.766	0 (0.0%)	-	0 (0.0%)	0.446
	Wave 2	0 (0.0%)		3 (0.7%)		3 (0.7%)		3 (0.7%)		0 (0.0%)		3 (0.7%)	
Anorexia	Wave 1	0 (0.0%)	0.4	0 (0.0%)	0.383	0 (0.0%)	0.543	0 (0.0%)	0.645	0 (0.0%)	0.53	0 (0.0%)	0.53
	Wave 2	2 (0.5%)		2 (0.4%)		2 (0.5%)		1 (0.2%)		2 (0.5%)		2 (0.5%)	
Diarrhoea	Wave 1	0 (0.0%)	0.55	0 (0.0%)	-	0 (0.0%)	-	0 (0.0%)	-	0 (0.0%)	0.53	0 (0.0%)	-
	Wave 2	1 (0.2%)		0 (0.0%)		0 (0.0%)		0 (0.0%)		2 (0.5%)		0 (0.0%)	
Chills	Wave 1	0 (0.0%)	-	0 (0.0%)	-	0 (0.0%)	-	0 (0.0%)	-	0 (0.0%)	-	0 (0.0%)	-
	Wave 2	0 (0.0%)		0 (0.0%)		0 (0.0%)		0 (0.0%)		0 (0.0%)		0 (0.0%)	
Decreased Appetite	Wave 1	0 (0.0%)	-	0 (0.0%)	-	0 (0.0%)	-	0 (0.0%)	-	1 (0.7%)	0.548	0 (0.0%)	-
	Wave 2	0 (0.0%)		0 (0.0%)		0 (0.0%)		0 (0.0%)		1 (0.2%)		0 (0.0%)	
Fever	Wave 1	0 (0.0%)	-	0 (0.0%)	-	0 (0.0%)	-	0 (0.0%)	-	0 (0.0%)	0.63	0 (0.0%)	-
	Wave 2	0 (0.0%)		0 (0.0%)		0 (0.0%)		0 (0.0%)		1 (0.2%)		0 (0.0%)	
Haemoptysis	Wave 1	0 (0.0%)	-	0 (0.0%)	-	0 (0.0%)	-	2 (1.3%)	0.204	1 (0.7%)	0.75	1 (0.7%)	0.548
	Wave 2	0 (0.0%)		0 (0.0%)		0 (0.0%)		1 (0.2%)		3 (0.7%)		1 (0.2%)	
Throat Pain	Wave 1	0 (0.0%)	-	0 (0.0%)	-	0 (0.0%)	-	0 (0.0%)	-	0 (0.0%)	-	0 (0.0%)	-
	Wave 2	0 (0.0%)		0 (0.0%)		0 (0.0%)		0 (0.0%)		0 (0.0%)		0 (0.0%)	

Fatigue at Contact 2 (Month 6) was reported by 9/153 (5.8%) Wave 1 and 19/444 (4.3%) Wave 2 participants (p = 0.39), indicating that the wave difference was not sustained. At Contact 3 (Month 12), fatigue fell further to 3/153 (1.9%) in Wave 1 and 5/443 (1.1%) in Wave 2 (p=0.57). At Contact 4 (Month 18), fatigue was numerically higher in Wave 1 than Wave 2 (4/153 (2.6%) vs. 3/443 (0.7%); p=0.123), but this did not approach significance. At Contacts 5 and 6 (Months 24 and 30), the numerical direction reversed - Wave 2 reported more fatigue than Wave 1 (Contact 5: 1/152 (0.7%) vs. 9/441 (2.0%), p=0.391; Contact 6: 1/152 (0.7%) vs. 10/441 (2.2%), p=0.336) - but, as with Contact 4, neither difference reached statistical significance, and the p-values at both contacts are considerably larger than at Contact 1, leaving little statistical support for treating these later fluctuations as a distinct clinical pattern.

Difficulty in breathing persisted at uniformly low rates across all six contacts in both cohorts (1.3%-1.8% in Wave 2; 0.6%-1.3% in Wave 1), without significant inter-wave differences at any contact (p=0.52-0.80). Dry cough was present in 0/155 (0.0%) Wave 1 and 10/446 (2.2%) Wave 2 participants at Contact 1 (p=0.06 - borderline, non-significant), declining to 1/153 (0.6%) vs. 7/444 (1.6%; p = 0.375) at Contact 2 and remaining low and non-significant through Contact 6.

Smell loss and taste loss - both near-universal COVID-19 hallmarks at acute onset - had nearly resolved by Contact 1: smell loss 1/155 (0.6%) vs. 1/446 (0.2%; p=0.43); taste loss 1/155 (0.6%) vs. 0/446 (0.0%; p=0.09). Both symptoms were absent in both waves from Contact 3 onwards, with the small residual numbers at Contacts 1-2 likely representing a genuinely slowly-resolving minority rather than reporting artefact. Rhinorrhoea was reported in 1/155 (0.6%) vs. 7/446 (1.6%; p=0.39) at Contact 1, and persisted at very low, non-significant rates throughout (p=0.18-0.66 at later contacts). Chest heaviness, myalgia, arthralgia, anorexia, diarrhoea, cough with expectoration, chills, decreased appetite, fever, haemoptysis, and throat pain were each present in negligible numbers across contacts, without significant inter-wave differences at any timepoint - most strikingly chills and throat pain, which were not reported by a single participant in either wave at any contact.

Sex, hospitalisation, and vaccination status as potential effect modifiers

Table 3 summarises unadjusted stratified comparisons exploring three potential effect modifiers of symptom persistence: sex, hospitalisation status during acute illness, and vaccination status. No symptom showed a significant difference by sex at any of the six contacts (all p>0.05), indicating that the inter-wave comparisons above are unlikely to be confounded by the sex imbalance in the overall cohort (60.1% men).

**Table 3:** Sex, Hospitalisation, and Vaccination Status as Potential Effect Modifiers of Symptom Persistence NS=Not significant (p>0.05). Cells reproduce the contact-wise significance pattern from the source presentation’s sub-group analysis slides (41–42); exact p-values are reported where the source specifies a precise value, and as “p<0.05” where the source reports only the threshold. These are unadjusted, univariate stratified comparisons and have not been adjusted for other covariates

Category	Symptom	C1	C2	C3	C4	C5	C6	Contacts with significant difference
A. Sex (Male vs Female)	All symptoms	NS	NS	NS	NS	NS	NS	No sex-based difference at any contact (all p>0.05)
B. Hospitalised (n=76) vs Non-hospitalised (n=525)	Anorexia	NS	NS	NS	p=0.02	NS	NS	Contact 4 (p=0.02)
	Chest Heaviness	p=0.03	NS	p=0.02	NS	NS	NS	Contacts 1 (p=0.03) & 3 (p=0.02)
	Difficulty in Breathing	p=0.004	p=0.001	p=0.02	NS	NS	NS	Contacts 1 (p=0.004), 2 (p=0.001) & 3 (p=0.02)
	All other symptoms	NS	NS	NS	NS	NS	NS	No significant association
C. ≥1 Vaccine Dose at Diagnosis vs Unvaccinated	Anorexia	p<0.05	NS	NS	NS	NS	NS	Contact 1 only
	Chest Heaviness	NS	NS	p<0.05	p<0.05	NS	NS	Contacts 3 & 4
	Difficulty in Breathing	p<0.05	NS	p<0.05	p<0.05	NS	NS	Contacts 1, 3 & 4
	Fatigue	p<0.05	p<0.05	NS	NS	NS	NS	Contacts 1 & 2
	Rhinorrhoea	p<0.05	p<0.05	NS	NS	NS	NS	Contacts 1 & 2
	All other symptoms	NS	NS	NS	NS	NS	NS	No significant association
D. Fully Vaccinated vs Not Fully Vaccinated	Arthralgia	p<0.05	p<0.05	NS	NS	NS	NS	Contacts 1 & 2
	Diarrhoea	NS	NS	NS	NS	p<0.05	NS	Contact 5 only
	Difficulty in Breathing	p<0.05	p<0.05	p<0.05	p<0.05	NS	NS	Contacts 1, 2, 3 & 4
	Fatigue	NS	NS	NS	NS	p<0.05	p<0.05	Contacts 5 & 6
	Haemoptysis	NS	NS	NS	p<0.05	p<0.05	NS	Contacts 4 & 5
	Rhinorrhoea	p<0.05	p<0.05	NS	NS	NS	NS	Contacts 1 & 2
	All other symptoms	NS	NS	NS	NS	NS	NS	No significant association

Prior hospitalisation during acute illness (n=76) was associated with a higher prevalence of difficulty in breathing at Contacts 1-3 (p=0.004, 0.001, and 0.02, respectively), chest heaviness at Contacts 1 and 3 (p=0.03 and 0.02), and anorexia at Contact 4 (p=0.02); no other symptom differed significantly by hospitalisation status at any contact. Incomplete vaccination at diagnosis was similarly associated with higher rates of fatigue and rhinorrhoea at the earliest two contacts, and with difficulty in breathing, chest heaviness, anorexia, arthralgia, haemoptysis, and diarrhoea at one or more individual contacts - in each case with fewer complaints in the more completely vaccinated comparison group, consistent with a partially protective effect of vaccination against symptom persistence, though we emphasise that these are unadjusted bivariate comparisons rather than estimates from a model adjusting simultaneously for hospitalisation, vaccination, age, and comorbidity; residual confounding between these correlated exposures (e.g., hospitalised patients being more likely unvaccinated, given the pre-vaccine timing of Wave 1) cannot be excluded from this stratified analysis alone.

## Discussion

This comparative community cohort - with six structured contacts assessing 17 post-COVID symptoms in 601 COVID-19 survivors from urban Hazaribagh, Jharkhand, across 30 months - provides some of the most granular longitudinal post-COVID symptom data available from a tier-two Indian city. The overarching and clinically most important finding is that post-COVID symptom burden is low and converges between first- and second-wave cohorts from the second contact onwards, with fatigue as the sole wave-discriminating symptom, and only at enrolment.

Fatigue: the most persistent and clinically significant post-COVID symptom

Fatigue at Contact 1 (enrolment) was the only symptom with a statistically significant wave difference across the entire follow-up period: 7.7% in Wave 1 versus 3.8% in Wave 2 (p=0.049). This finding requires careful contextualisation. Because enrolment for the two waves occurred at different points relative to the underlying date of infection - first-wave participants were necessarily enrolled later in calendar time relative to their own infection than second-wave participants, given the staggered timing of the two waves and the shared enrolment window - a differential lag between infection and the Contact 1 assessment may have consolidated a chronic fatigue phenotype in a subset of Wave 1 survivors who had already passed their initial acute recovery window by the time of interview. This lag is an unavoidable methodological consequence of the sequential wave design and likely explains at least part of the higher fatigue prevalence in Wave 1 at Contact 1.

By Contact 2 (Month 6), the fatigue difference was no longer statistically significant (5.8% vs. 4.3%; p=0.39), confirming convergence. At Contacts 4-6, fatigue prevalence fluctuated numerically between the two waves without approaching significance at any point (p=0.123, 0.391, and 0.336 respectively); given these consistently large p-values, weak absolute differences in single-digit counts, and the absence of any corrected threshold for the 17 symptoms and six contacts examined, we have deliberately avoided characterising this later fluctuation as a distinct “cross-over” phenomenon or as evidence of a genuine long-COVID subgroup, since the data do not support that inference; the more parsimonious explanation is sampling noise around a uniformly low prevalence in both cohorts from Month 6 onwards.

Pooled fatigue prevalence in long-COVID meta-analyses is estimated at 58% [6], substantially higher than our 7.7% at Contact 1. This gap reflects fundamental sampling differences: most meta-analysed cohorts are hospital-based and symptom-enriched, while our systematic IDSP registry sampling captures the full community spectrum, including the mild-to-moderate majority. The UK CO-FLOW study - a multicentre post-discharge cohort - documented fatigue in over 50% at 12 months [11], but restricted to hospitalised patients who inherently carry greater residual organ dysfunction. Within community-based cohorts, Tenforde et al.'s Morbidity and Mortality Weekly Report (MMWR) outpatient telephone study found that 35% had not returned to usual health by two to three weeks [12], though this early timepoint captures acute-phase resolution rather than genuine long-COVID.

Convergence of post-COVID trajectories: a reassuring finding with practical implications

Beyond fatigue at Contact 1, no symptom at any of the remaining five contacts showed a statistically significant wave difference. This convergence is biologically plausible. While the acute presenting phenotype of COVID-19 is shaped by variant-specific properties, the post-acute trajectory is governed by common host-level mechanisms - immune dysregulation, mitochondrial dysfunction, autonomic instability, and psychosocial sequelae - that are largely independent of the infecting variant [13]. Nalbandian et al.'s comprehensive review of Post-Acute Sequelae of SARS-CoV-2 (PASC) documented these overlapping mechanisms across survivors of all variants and disease severities [13]. The practical implication remains that a single, uniform post-COVID follow-up protocol is reasonable for survivors of both waves; variant-specific differentiation of long-term management pathways is not supported by this evidence. We note, however, that this conclusion describes the comparative trajectory between two survivor groups, and - in the absence of a SARS-CoV-2-negative reference group - cannot itself establish what proportion of the residual symptom burden in either wave reflects true post-COVID pathology versus background community symptom prevalence unrelated to COVID-19.

Symptom-specific trajectories

Difficulty in breathing persisted at uniformly low but consistent levels across all six contacts in both cohorts (~0.6-1.8% per wave per contact), without significant inter-wave differences, though - as detailed above - it was significantly more common among previously hospitalised participants at the first three contacts. This small persistent subgroup with ongoing dyspnoea likely represents genuine post-COVID pulmonary parenchymal or airway pathology, consistent with the finding of persistent reduced diffusion capacity in a subset of post-COVID survivors documented by Huang et al. [14]. These individuals may benefit from targeted respiratory assessment and pulmonary rehabilitation referral.

Smell loss and taste loss resolved almost completely by Contact 1, with only one to two patients per wave retaining either symptom, and complete resolution from Contact 3 onwards. This rapid recovery is consistent with Morin et al.'s French inpatient cohort, which documented near-complete resolution of olfactory dysfunction at four months in the majority [15]. The minority with persistent anosmia beyond 12 months constitutes a clinically distinct subgroup warranting specialist otorhinolaryngeal assessment and potential olfactory training.

Dry cough resolved rapidly after the acute phase with no significant inter-wave differences at any follow-up contact; the borderline trend at Contact 1 (0.0% vs. 2.2%; p=0.06) was transient and non-significant by Contact 2, supporting the concept that variant-specific upper-airway symptoms are acute-phase phenomena that do not translate into long-term wave-specific differences in post-COVID cough burden. Rhinorrhoea, myalgia, arthralgia, chest heaviness, anorexia, diarrhoea, cough with expectoration, decreased appetite, and fever each persisted in very small absolute numbers throughout, without significant inter-wave differences; chills and throat pain were not reported by any participant in either wave at any contact. These minimal residual symptoms likely represent coincidental background illness rather than genuine post-COVID manifestations at these late timepoints, given the low absolute numbers and absence of wave specificity. Haemoptysis appeared at low rates from Contact 4 onwards in both waves; while not statistically significant at any contact, any new haemoptysis in a post-COVID patient warrants clinical evaluation to exclude pulmonary thromboembolic sequelae, bronchiectasis, or other pathology.

Effect modification by hospitalisation and vaccination status, not sex

The absence of any sex-based difference in symptom persistence at any contact (Table 3) argues against sex as a meaningful confounder of the inter-wave comparisons reported above despite the cohort's male predominance (60.1%); this directly addresses a concern raised during peer review. By contrast, prior hospitalisation and incomplete vaccination status were each associated with a small number of respiratory and constitutional symptoms at specific contacts, predominantly in the first 12-18 months of follow-up - a pattern consistent with greater initial organ-system insult among the more severely affected (hospitalised) and less-protected (incompletely vaccinated) subgroups. These associations should be read as exploratory and hypothesis-generating rather than confirmatory: hospitalisation, vaccination status, wave, and age are correlated with one another in this cohort (Wave 1 was entirely pre-vaccine and had a higher hospitalisation rate than Wave 2), and a multivariable model capable of disentangling their independent contributions - for example, generalised estimating equations or mixed-effects logistic regression incorporating wave, hospitalisation, vaccination, age, and comorbidity simultaneously, appropriately accounting for the repeated-measures structure across the six contacts - was beyond the scope of the descriptive, contact-wise dataset available for this analysis, and is recommended as a priority for future analysis of this cohort.

Comparison with published Indian data

Published longitudinal community post-COVID data from Jharkhand are very limited. Kumar et al.'s prospective cohort from the All India Institute of Medical Sciences (AIIMS) Deoghar - the geographically most relevant comparator - reported 78.7% of non-hospitalised patients still symptomatic at 18 months via telephone interview [9]. This is markedly higher than our figures at the equivalent Contact 3 (Month 12: 1.9% in Wave 1; 1.1% in Wave 2). The discrepancy likely reflects a self-selected symptomatic cohort in Kumar et al. versus our registry-based systematic sampling of all confirmed positives irrespective of ongoing symptoms.

The Delhi multi-district community cohort (n=410) reported a long-COVID prevalence of 22% at three to six months [16], higher than our ~9% at Contact 1. Differences in infecting variant (Omicron era in the Delhi study), long-COVID definition, and follow-up design (single contact vs. our serial structured contacts) likely account for this disparity. Our structured 30-month cohort represents one of the longer published systematic community follow-ups of post-COVID symptoms from this region of India.

Strengths and limitations

Strengths

Prospective design with six structured contacts over 30 months; community-based systematic sampling from IDSP registers capturing the full-disease severity spectrum; large sample (n=601) spanning two wave cohorts; standardised questionnaire applied consistently; minimal attrition (<1% cumulative loss by Contact 6); direct referral pathway for symptomatic participants through SBMCH; and exploratory stratified analysis of sex, hospitalisation, and vaccination status as potential effect modifiers.

Limitations

This is a comparative study of two survivor groups rather than a true cohort study with an unexposed control arm, restricting inference to between-wave comparison; the two cohorts differ substantially in size (155 vs. 446) as a direct consequence of the underlying registry sizes, which widens the confidence interval around Wave 1 estimates relative to Wave 2 at low-prevalence contacts; the differential enrolment lag between wave cohorts at Contact 1 is an important methodological limitation, as it may have inflated fatigue prevalence in Wave 1 at that contact; symptom data are self-reported and susceptible to recall bias, without objective clinical quantification (spirometry, olfactometry, echocardiography); duration of smoking and smokeless tobacco use was not captured, precluding analysis of dose-duration effects on respiratory symptoms; the dataset available for this analysis recorded aggregate per-contact, per-wave symptom counts rather than a participant-level longitudinal file, which precluded formal intra-cohort longitudinal modelling (e.g., generalised estimating equations or mixed-effects regression) across the six contacts, multivariable adjustment for confounders, and calculation of confidence intervals; no correction for multiple comparisons across 17 symptoms and six contacts was applied, so the single significant finding (fatigue, Contact 1) should be interpreted with appropriate caution; and the study is restricted to urban Hazaribagh, limiting generalisability to rural and tribal Jharkhand.

## Conclusions

In this prospective comparative study of 601 COVID-19 survivors from urban Hazaribagh, Jharkhand, post-COVID symptom burden was consistently low and showed no statistically significant wave-specific difference at any contact beyond the first. Fatigue was the sole exception - significantly more prevalent in first-wave participants at Contact 1; by Contact 2, even this difference had resolved. Sex showed no association with symptom persistence at any contact, whereas prior hospitalisation and incomplete vaccination status were each associated with selected respiratory and constitutional symptoms in the earlier part of follow-up, in unadjusted stratified analyses.

These findings support three actionable conclusions for clinical practice: (1) a single, uniform post-COVID follow-up protocol is reasonable for survivors of both waves, since no consistent variant-specific differentiation in long-term management emerged from this comparative analysis; (2) fatigue, and to a lesser extent difficulty in breathing, should remain the primary focus of clinical attention in post-COVID follow-up in this community setting, with closer attention to participants with a history of hospitalisation or incomplete vaccination; and (3) systematic post-COVID assessment embedded within existing primary healthcare infrastructure in Jharkhand, beginning at or soon after the index illness, would be sufficient to identify the small subgroup with persistent symptoms across both wave cohorts.
